# Meal support intervention for eating disorders: a mixed-methods systematic review

**DOI:** 10.1186/s40337-024-01002-2

**Published:** 2024-04-22

**Authors:** Aleshia Ellis, Kerri Gillespie, Laura McCosker, Carly Hudson, Gideon Diamond, Tawanda Machingura, Grace Branjerdporn, Sabine Woerwag-Mehta

**Affiliations:** 1 Gold Coast Hospital and Health Services, 1 Hospital Boulevard, Southport, QLD 4215 Australia; 2 Bond University, 14 University Drive, Robina, QLD 4226 Australia

**Keywords:** Meal support, Intervention, Systematic review, Eating disorders, Multidisciplinary, Anorexia, Bulimia

## Abstract

**Objective:**

Mealtimes are a period of heightened distress for individuals with eating disorders. Patients frequently display maladaptive coping strategies, such as hiding food and using distraction techniques to avoid eating. The aim of this systematic review is to evaluate the evidence for meal support interventions as a first-line intervention for eating disorders.

**Method:**

Six databases were systematically searched in January 2024. Papers including patients with an eating disorder, and meal support or meal supervision, were examined. Quality appraisal was conducted.

**Results:**

Ten studies met inclusion criteria. Meal support was conducted individually and in group settings. Two studies examined the practical or interpersonal processes of meal support. Carers and trained clinicians implemented meal support. Individuals across the lifespan were examined. Settings included inpatient units, community clinics, and the home. Studies were heterogeneously evaluated with retrospective chart audits, pre- and post- cohort studies, semi-structured interviews, video analysis, and surveys.

**Discussion:**

Meal support intervention is potentially suitable and beneficial for patients of various age groups and eating disorder diagnoses. Due to the lack of consistent approaches, it is apparent there is no standardised framework and manualised approach. This highlights the need for the development of a co-designed approach, adequate training, and rigorous evaluation.

**Supplementary Information:**

The online version contains supplementary material available at 10.1186/s40337-024-01002-2.

## Abstract (plain language summary)

Previous research indicates that meal support may be potentially beneficial as an independent intervention in the treatment of eating disorders, but inconsistent approaches and a lack of standardization make evaluations challenging. The current study aims to provide an overview of current meal support interventions, how they are implemented, and their impacts on health outcomes and hospital admissions in people experiencing an eating disorder. Gaps in current knowledge and research highlight the need for further investigation, and the development of a co-designed approach, adequate training, and rigorous evaluation.

**Supplementary Information:**

The online version contains supplementary material available at 10.1186/s40337-024-01002-2.

## Background

Eating and drinking are fundamental and life sustaining activities. Eating is a learnt behaviour, contrary to thoughts it is innate [1]. Findings from a large number of studies indicates that eating meals together as a family is associated with favourable dietary patterns and improved physical and psychosocial outcomes in children, including fewer rates of obesity, decreased risk for eating disorders, and improved academic achievement [2]. An inverse correlation between family meal frequency and weight-control behaviour, binge eating, and chronic dieting, for females has been reported [3–5].

For individuals with eating disorders, meal times are a period of heightened distress [6]. Negative emotions, such as fear, anxiety, disgust, irritability, anger and depression increase during mealtimes [7–10]. To cope with these feelings patients frequently display maladaptive coping strategies, such as avoiding eating certain foods, avoiding eating with others, hiding food, covertly exercising, and using distraction techniques to avoid eating [7, 11, 12].

Whilst a strong predictor of clinical outcome is eating behaviour, directly or indirectly related to weight restoration [13, 14], support during meal times is a critical and effective component of nutritional rehabilitation [15]. There is no consistent approach consensus or guideline on how to best support an individual with disordered eating behaviours during meal times [10, 16, 17].

The most researched and utilised form of meal support is the family meal, a component of Maudsley Family Based Therapy (MFBT), Family Therapy (FBT) and Multifamily Therapy (MFT), the current gold standards of treatment for children with anorexia nervosa and bulimia nerviosa. The family meal is typically conducted during the first phase of treatment. The focus, approach and number of family meals conducted is highly variable with some models emphasising normalising eating, whilst others focus on the improvement of family relations and interactions [18, 19].

Clinicians and personal supports (including families and caregivers) of those with eating disorders report mealtimes as being distressing, putting caregivers and clinicians at risk of emotional burn out [20–23]. The family meal has been described as particularly challenging and experienced by some as a cause for therapeutic breakdown and subsequent disengagement of families from therapy. Hence, adherence to this element of treatment is poor, with approximately 40.0% of clinicians reportedly not pursuing a family meal during MFBT/FBT [15, 24, 25]. An investigation of therapist perspectives on MFBT and FBT found highly inconsistent implementation of the family meal as a standard part of treatment, with only 25.0% conducting a meal on a regular basis [26]. Limited training in these specific therapeutic components, reluctance to view them as part of their therapeutic role, and a sense of intimidation and anxiety were reported reasons for the lack of adherence to the treatment protocol. Over a third (36.5%) of clinicians who reported using FBT rarely include the family meal in their practice [25]. In a study that investigated carers’ views on single and dual-family treatment for AN, some caregivers viewed the family meal as beneficial, but many perceived it as anxiety provoking or ‘‘false” [24]. A recent systematic review concluded that the usefulness of family meals in family therapy for eating disorders is not clear [27]. On the other hand, emerging evidence for alternative approaches to family meals, such as direct advice or DVDs, have shown to be acceptable and effective in inducing weight gain and reducing caregiver distress [28–30].

Inmproving the support provided at mealtimes is a core component of ED treatments, that aim to not only normalise weight, but also nromalise meal eating behaviours, and progress patients toward independence [15, 31]. Previous research into the efficacy of meal support has predominantly investigated techniques that are part of a larger, family-based treatment model. A dearth of research relates to the potential benefit of meal support as a first-line intervention. Treatment Modalities to improve support during meal times are rooted in family therapy and have been used predominantly in child and adolescent populations. Little is currently understood about the impacts of meal support in adults compared to younger populations. We have therefore included all age groups in this review in an attempt to further understand the impacts of meal support in different age groups, and determine any differential effects. The differential impacts of meal support for different diagnoses (specifically AN compared to ARFID in younger cohorts) is also poorly understood and requires further investigation. The aim of this systematic review is to investigate whether meal support is being used as a first-line intervention, how these interventions are being implemented and for whom, and the characteristics of these interventions and where they take place. A secondary aim of the review is to evaluate the evidence for meal support interventions as a first-line treatment for eating disorders by identifying the outcomes of these interventions in terms of patient health and satisfaction of patients, parents and staff. While “meal support” and “meal supervision” was used interchangeably across studies, the term “meal support” will be used for the purpose of this review.

## Method

This systematic review was conducted according to the 2020 PRISMA reporting guidelines for Systematic Reviews and Meta-Analyses [32]. The review protocol was registered on the PROSPERO database (registration no. CRD42022311374). A narrative synthesis was conducted where papers were too heterogeneous or contained too little data to conduct meta-analysis.

### Research questions

The systematic review will provide an exploratory investigation of the evidence for meal support interventions as a first-line treatment, by answering the following research questions:

1. What are the characteristics of existing, first-line, meal support interventions described in the literature?
2. What are the outcomes of first-line meal support interventions, in terms of patient health (weight gain, food consumption, length of stay)?
3. What are the qualitative outcomes of first-line meal support interventions, in terms of patient, parent, or staff satisfaction and opinions.

### Search strategy

Search terms were chosen after investigation of the literature, and consultation with eating disorder researchers and clinicians. Preliminary searches were conducted to identify the optimal search strategy and to eliminate overly broad terms and abbreviations that retrieved excessive numbers of unrelated articles (such as AN, ED, and BED). The search strategy, outlined in Table 1, contained keywords and MeSH terms relevant to meal support and meal supervision for people with a diagnosis of an eating disorder. Six online databases were systematically searched in December 2021: CINAHL, EMBASE, PsycInfo, PubMed, Scopus, and Web of Science. A manual, hand search of reference lists of included papers, and of related systematic reviews, was also conducted.

**Table 1:** Search strategy

Concept Category	Search Terms
Eating disorders	(“Eating Disorder*” OR “Eating Disorders”[MeSH Terms] OR Anorexia OR Bulimia OR BN OR “Binge Eating Disorder” OR “Eating Disorder Not Otherwise Specified” OR EDNOS OR “Avoidant Restrictive Food Intake Disorder” OR ARFID OR purging OR “binge eating”)
Meal support interventions and treatments for eating disorders with a meal support component	AND (“Family-Based Therapy” OR “Family-Based Treatment” OR FBT OR Maudsley OR FBMT OR “Cognitive behavior therapy for eating disorders” OR CBTE OR CBTED OR “Meal Support” OR “Meal supervision” OR “Practical Eating” OR “Meal Intervention” OR “Practical Food Group*” OR mealtime OR meal* OR “multifamily therapy” OR MFT OR MFBT)

### Selection criteria

Papers were included based on the following criteria:

- Patients had a diagnosis of an eating disorder [anorexia nervosa, bulimia nervosa, binge eating disorder, avoidant and restrictive food intake disorder (ARFID)].
- Meal support or meal supervision was used.

Papers with no empirical data, or which were not available in English were excluded. There were no restrictions on the type of study setting in which the meal support intervention occurred (e.g., inpatient, ambulatory clinics, home, public and private); type of methodology employed (e.g., qualitative, quantitative or mixed methods); or age of the participant.

### Screening process

Duplicate papers were removed from the original yield of the databases. Titles and abstracts were reviewed by two independent raters based on the inclusion criteria. The full-texts of remaining articles were also independently reviewed by two raters. In cases of disagreement, the suitability of the article was discussed and consensus reached, or another researcher mediated the discussion to make a final determination about the article’s inclusion.

### Quality assessment

The McMaster Quantitative and Qualitative Assessment Tools, and the Mixed-Methods Appraisal Tool (MMAT) [33] were used to appraise the quality of included articles. The MMAT is a 19-item checklist designed to concomitantly appraise the methodological quality of quantitative, qualitative and mixed methods studies. Studies were independently assessed by two raters and the findings were compared. In cases where there was discrepancy, results were discussed until a consensus was reached.

## Results

### Study selection

**Fig. 1:**
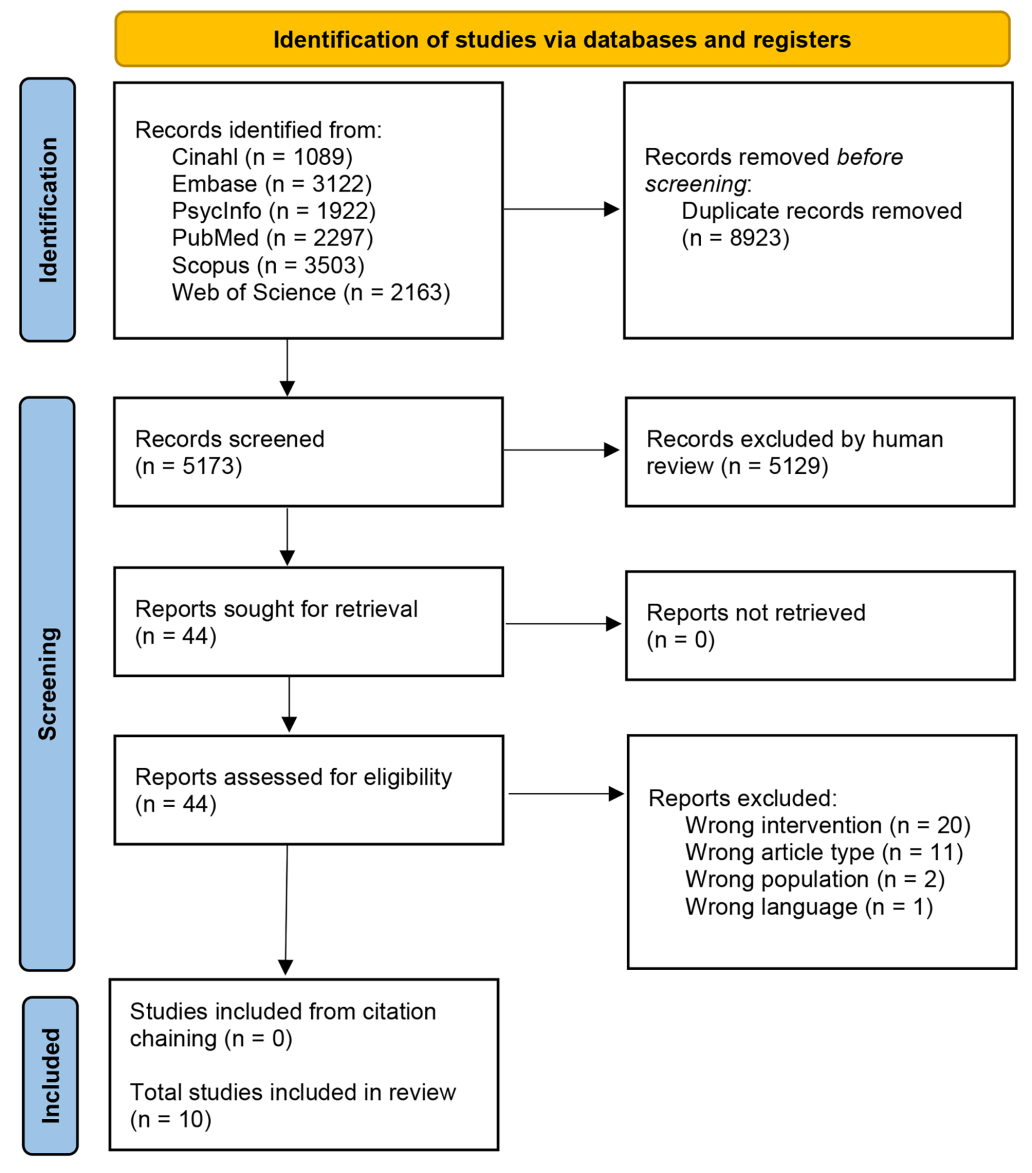
PRISMA flow diagram

14,096 studies were identified through database searching. Once duplicates were removed, the title and abstracts of 5,173 studies were screened, excluding 5,129 studies. Forty-four full-texts were then reviewed for eligibility, resulting in 34 articles being excluded. Reasons for exclusion were that studies were not focused on meal support as a first-line intervention (*n* = 20), were a conference abstract, poster, dissertation or other non-eligible paper type (*n* = 11), included the wrong patient population (*n* = 2), or not available in English (*n* = 1). A PRISMA flowchart of the study screening and selection process is presented in Fig. 1.

### Methodological quality of studies

Quality assessment results are displayed in Supplementary Tables 1, 2, and 3. Purpose, background literature, study design and type, results’ statistical significance, analyses clinical importance and conclusions were described well for quantitative studies (*n* = 4) [15, 34–36]. Two studies did not include one or more of the following details: sample size justification, validity of outcome measures, description of the intervention, contamination and cointervention avoided, and drop-out reporting [35, 36]. Qualitative studies (*n* = 4) [37–40] mostly met the quality appraisal criteria except for two studies [39, 40] which did not report, or provided inadequate detail, for one or more of the following aspects: theoretical perspective, obtaining informed consent, identifying assumptions and biases of the researcher and reporting on the decision trail. Kells (2013) reported better outcomes in patients who received meal support compared to patients who did not receive meal support; however, it is unclear whether patient characteristics, severity of illness, length of diagnosis, and physical compromise were comparable to the intervention group. Mixed-methods studies (*n* = 2) [10, 41] tended to meet the quality appraisal criteria except for not integrating quantitative and qualitative results, and addressing inconsistencies in the results between these two components.

### Study characteristics

Quantitative (*n* = 4) [15, 34–36], qualitative (*n* = 4) [37–40], and mixed-methods design (*n* = 2) [10, 41] were identified. Specific methodologies included retrospective chart audits [15, 35, 36], a pre- and post- cohort study [34], semi-structured interviews [10, 39, 40], video analyses [37, 38], and surveys [10, 41].

Most studies (*n* = 7) were conducted within an inpatient specialist eating disorders unit [10, 15, 35–38, 40]. A private eating disorders clinic [34], child and adolescent public tertiary mental health community service [41], and home were also identified [34, 39]. The sample size ranged between 9 and 56 participants.

### Study participants

Study participants included patients diagnosed with an eating disorder (anorexia nervosa, bulimia nervosa, ARFID, binge eating disorder) (*n* = 5) [15, 34–36, 40] and in two, linked studies, a mixed group of patients with an eating disorder and some patients with a diagnosis of disordered eating not meeting diagnostic threshold [35, 36] receiving meal support; clinicians providing meal support (*n* = 4) [10, 37–39]; and parents and carers of patients with eating disorders (*n* = 1) [41].

#### Patient characteristics

As seen in Table 2, most studies (*n* = 7) examined patients with a primary diagnosis of anorexia nervosa [15, 35–38, 40, 41]. Patients with an eating disorder not otherwise specified (EDNOS) were investigated in two studies [36, 41]. Diagnoses of bulimia nervosa [41] and ARFID [34] were included in one study each. One study also included in their cohort some patients who did not meet the diagnostic threshold fo an eating disorder diagnosis [36]. Average Body Mass Index (BMI) ranged between 14 and 16.1 in the three studies that reported BMI [35, 36, 38].

**Table 2:** Patient characteristics

Author/s	Article type	*N*	Primary Diagnosis				Mean Body Mass Index (BMI) at Admission (kg/m^2^)	Age (years)			Sex
			Anorexia Nervosa	Bulimia Nervosa	EDNOS	ARFID		Range	*M*	*SD*	
1. Couturier and Mahmood (2009)	Quantitative	21	✓				NR	11.7–17.7	15.1	1.9	F (90%)
2. Kells et al. (2013)	Quantitative	52	✓				15.9	9.7–21.7	15.9	2.5	F (96%)
3. Kells et al. (2017)	Quantitative	Control = 52 Intervention = 56	✓		✓		15.9 16.1	NR	17.4 14.8	3.6 2.3	F (96%) F (89%)
4. Taylor et al. (2021)	Quantitative	26				✓	NR	2–13	6	NR	F (15%)
5. Beukers et al. (2015)	Qualitative	9	✓				NR	12–18	NR	NR	NR
6. Hage et al. (2015)	Qualitative	NA	✓				14–15	16+	NR	NR	NR
7. Long et al. (2012a)	Qualitative	12	✓				NR	17.4–29.5	22.08	3.74	F (100%)
8. Watt and Dickens (2018)	Qualitative	NA	NA				NA	NA	NA	NA	NA
9. Cairns et al. (2007)	Mixed methods	40	✓	✓	✓		NR	13–18	15.1	NR	NR
10. Long et al. (2012b)	Mixed methods	NA	NA				NA	NA	NA	NA	NA

Note. NR = not reported, NA = not applicable, F = female

Most (*n* = 6) studies included adolescents between 12 and 18 years old [15, 35, 36, 41], staff and patients at facilities that catered for adolescents aged 12 to 18 [37], or staff that treated adolescents 16 and over [38]. Two studies included patients under 12 years [34, 35]. Three studies included patients over the age of 18 [35, 36, 40]. One study included staff members for facilities treating patients of all ages [10]. Three studies involved exclusively [40] or predominantly [15, 35] female patients, and one study focused on mostly male patients [34]. Studies that did not specify gender focused on study characteristics and / or included staff members as participants.5)

#### Clinician characteristics

Three studies, reported on clinicians of varying professional backgrounds delivering meal support: including nursing staff; clinical support staff; consultant psychiatrists; social workers, and; child welfare officers [37–39].

#### Intervention characteristics

As seen in Table 3, staff to patient ratios varied across inpatient units starting from 1:1 [10, 34, 39] and ranging up to 1:10 [10]. Most (*n* = 9) studies provided meal support by a trained clinician [10, 15, 34–40].

**Table 3:** Description of meal support (MS) and study outcomes

Author/s	Article type	Study Setting	Study Country	Aim	Number of participants, videos, units	Description of Meal Support (MS)	Outcomes
1. Couturier and Mahmood (2009)	Quantitative	Child and adolescent inpatient psychiatric unit	Canada	Retrospectively assess whether the implementation of MS had an impact on the use of nasogastric feeding in AN patients	Group1: Pre-MS implementation: 12 children (< 18y) with AN restricting or binge purging type Group2: Post-MS implementation: 9 children (< 18y) with AN restricting or binge purging type	• Staff provided supervision for meals and snacks • Staff provide emotional support, whilst being directive about required food consumption.	• Incidence of nasogastric feeding decreased (*p* < 0.02) • Weight on admission was lower in the post MS group (*P* < 0.03) • No difference was found in Weight change, rate of change and discharge weight length of stay, and readmission rate
2. Kells et al. (2013)	Quantitative	Tertiary children’s hospital	United States of America	Examine the effect of MS on outcomes during inpatient medical hospitalization	Control: 52 patients with diagnoses of ED NOS, AN, both restrictive and purging types, and patients who are pre-diagnosis	• MS was described as a modification of MFBT, - • MS was not part of standard practice. • MS was provided by staff	• Mean weight increased • length of stay decreased • overnight bradycardia decreased.
3. Kells et al. (2017)	Quantitative	Tertiary children’s hospital	United States of America	Examine the effect of standardized MS on weight gain, length of stay, vital signs, electrolytes, and use of liquid caloric supplementation in hospitalized adolescents and young adults with restrictive eating disorders	Intervention: 56 patients with diagnoses of EDNOS, AN, both restrictive and purging types, and patients who did not meet diagnostic criteria	• MS was described as a modification of MFBT • MS started at admission as part of standard practise • MS was provided by staff	• No significant difference between groups was found
4. Taylor et al. (2021)	Quantitative	Private clinical practice	Australia	• Examined whether treatment gains were maintained when trained parents continued the programme at home and during meals out	26 children and their parents	• MS was individualised and targeted to the needs of the patients. • MS involved changing the mealtime environment, providing incentives for appropriate food intake, setting realistic and achievable mealtime goals, persistent presentation of food, and teaching practical eating skills (e.g., chewing, biting, using utensils).	• Food intake increased. • Variety of foods increased (mean = 92 different foods) • Decrease of inappropriate mealtime behaviours. • Treatment gains were maintained during follow-up at a mean of 2.3 years. • Parental satisfaction and treatment acceptability were reported high.
5. Beukers et al. (2015)	Qualitative	Specialist inpatient eating disorders unit	The Netherlands	Identify interventions used by health professionals in a specialist eating disorder centre to restore normal eating behaviour for adolescents diagnosed with anorexia nervosa	8 health-care professionals trained in diagnostics, motivational aspects, CBT, FBT, (relapse) prevention, and dietetics were videotaped during mealtimes	• MS was provided in a group setting with patients at various stages of recovery. • Staff provided emotional support and direction to patients and patients who were further along in the recovery journey provided peer support.	• MS aspects identified included: monitoring and instructing, encouraging, and motivating, supporting, and understanding, educating.
6. Hage et al. (2015)	Qualitative	Inpatient eating disorders unit	Norway	Determine the structure of a meal, revealing the operating scripts.	22 staff members (nurse, social workers, child welfare officers) 40 meals were filmed, 10 of each meal type (breakfast, lunch, dinner, and evening meal)	• Patients were supervised by staff during mealtime. Food intake was monitored over a 30-minute period. If meals were not completed within this time, a nutritional replacement was offered.	• Video recording analyses identified 3 mealtime phases: pre-eating (serving and positioning), eating (division of labour and dialogue), and completion (end of meal preparations).
7. Long et al. (2012a) ^	Qualitative	Public NHS and private eating disorder services	United Kingdom	Investigate in-patient perceptions of mealtimes on eating disorders units.	12 patients with AN	• Patients participated in group meals in the inpatient unit for a minimum of 2 weeks, • Staff was eating alongside patients while supervising food intake.	• Interviews relating to patient experience of mealtimes revealed Three themes were identified: mealtime delivery (logistical factors influencing meals), individual outcomes (cognitions, emotions, behaviours, and physical sensations during meals), mealtime characteristics disengagement, perceived battlegrounds, and a desire for involvement in more decision making at mealtimes)
8. Watt and Dickens (2018)	Qualitative	Child and Adolescent Mental Health Services	Scotland	Explore mental health clinicians’ perspectives on community mealtime management with children and adolescents diagnosed with an eating disorder	6 mental health clinicians with experience of delivering or referral for the intervention completed semi structured interviews	• MS was delivered by a specialist intensive community team (ICT) in the home environment. • Family prepared meals in accordance with the agreed diet plan • Meal duration was set at max 30 min. • ICT clinicians provided support and supervision during a meal.	• Interviews relating to clinician’s experience in delivering MS revealed 3 themes: technical and emotional aspects mealtime management, and the combination of food intake and nutritional supplements.
9. Cairns et al. (2007)	Mixed methods	Eating disorder treatment centre, British Columbia Children’s Hospital	Canada	Evaluate the helpfulness of the contents of a video and manual for training parents and caregivers in providing meal support for eating disordered youth.	40 self-report questionnaires consisting of closed and open-ended questions were collected from parents or caregivers	• A DVD and manual were provided to parents and carers to introduce concepts of MS, help parents and caregivers empathise and outline support strategies.	• DVD and manual resources were found beneficial in providing insight into emotional processes of eating disorder patients and teaching practical MS strategies. • Resources were reported as usefulness dependent upon the patient’s stage in recovery.
10. Long et al. (2012b)	Mixed methods	NHS and specialist eating disorder units	United Kingdom	Study 1: Assess the current mealtime practices within UK eating disorders units. Study 2: Investigate staff perspectives of these mealtimes, including their involvement and understanding of patients’ experience	Study 1: 6 (out of 22 identified specialist eating disorder inpatient units Study 2: 16 staff members of 3 specialist eating disorder inpatient units participated in an interview	• Patients’ meals were supervised by staff • Staff could opt to eat with the patients.	Study 1: MS practises varied within and between units. Study 2: Three themes were identified: preparation, roles during mealtime, and barriers.

Note. NHS (National Health Service) is the public health service within the United Kingdom

^The term Mealtime practises was used

Anorexia Nervosa (AN), Bulimia Nervosa (BN), Avoidant Restrictive Food Intake Disorder (ARFID), Eating Disorder Not Otherwise Specified (EDNOS), Cognitive Behavioural Therapy (CBT), Maudsley Based Family Therapy (MFBT), Meal Support (MS)

Two studies examined the practical [38] or interpersonal [37] processes of meal support delivered within inpatient units. Practical processes consisted of three phases: preparatory (meals are served, and patients are asked to be seated at the table with their food), eating (patients and inpatient staff sit at the table and eat their meals, with support from the staff), and post-meal (patients finish eating and leave the dining room with the staff) [38]. Interpersonal aspects of meal support included: monitoring food intake, providing mealtime instruction, motivating and encouraging patients to complete the meal, expressing support and understanding, and providing psychoeducation [37].

The length of time of mealtimes varied from 30 min [34, 37, 38], to 60 min [10]. Supervised rest period immediately after the meal ranged from 15 to 60 min [10, 15, 38, 39]. While it is common practice in eatig disorder treatments (e.g., CBT) to use graduated exposure to ‘fear-foods’ in ARFID and AN, none of the articles described implementing a graduated approach to meal supervision.

Aesthetics of the dining room (e.g., size, shape, and setting of the dining table), timing of the meals to avoid delays, and consistency in approach were important aspects identified to alleviate distress in patients [10, 40]. Familiarity with the clinicians and having a standardised training approach were facilitators of meal support effectiveness [39]. Furthermore, clinicians providing empathic emotional support during mealtimes, such as encouraging patients to continue eating and reducing feelings of anxiety [10, 15], whilst also being assertive and firm around food consumption [10, 15, 37] was reported being effective. Distraction techniques utilised and identified as helpful included discussing unrelated topics, employing breathing techniques, playing games, and watching television or listening to radio [10, 15]. Staff, however, were uncertain around appropriate topics to discuss [10] and voiced that distraction could prolong eating time [40]. Peer support was found to be beneficial to instil hope that recovery was possible [37]. Interviews relating to patient experience of mealtimes revealed three themes: mealtime delivery (logistical factors influencing meals), individual outcomes (cognitions, emotions, behaviours, and physical sensations during meals), mealtime characteristics disengagement, perceived battlegrounds, and a desire for involvement in more decision making at mealtimes [40].

The importance of training parents and carers in providing meal support post-discharge was acknowledged in three studies [34, 39, 41]. Rigorous training of parents and carers of patients with ARFID in the provision of meal support at home was shown to be a successful alternative to long-term eating disorder outpatient treatment. Parental satisfaction and treatment acceptability were reported high and treatment gains were maintained during follow-up at a mean of 2.3 years [34].. Distribution of a manual and DVD for psychoeducation and meal support training was described as effective and valuable to parents and carers [41].

### Quantitative outcomes

Three studies found positive outcomes with increased weight gain and fewer episodes of bradycardia [35], shorter hospital stays [36], reduced incidence of nasogastric feeding [15], and reduced incidence of inappropriate mealtime behaviours [34]. However, Kells and colleagues [35] found that meal supervision was associated with longer hospital stays. Two studies also found no differences between supervised (meal support) and non-supervised meals in terms of weight gain [15, 36], electrolytes or vital signs [36], length of hospital stay [15], or rate of readmission [15]. All of these studies were conducted in child, adolescent, and young adult cohorts.

### Experiences with meal support

Patients suggested simulating post-discharge meals, and reported that staff who eat alongside them and provide both empathic support and understanding of negative cognitions was important [10]. Parents and caregivers were satisfied with resources as it empowered them and increased their understanding; however, the stage of readiness of the patient needed to be considered [41]. Two studies explored clinicians’ experiences and feedback with regards to delivering meal support and supervision, suggesting that training in a uniform approach and debriefing sessions supported patient outcomes [37, 39].

## Discussion

This literature review identified ten studies, that examine the role of meal support as a standalone intervention for eating disorders, using quantitative, qualitative and mixed methods approaches. Most studies met quality appraisal criteria with average ratings. Due to small numbers and heterogeneity in design and methodology comparisons between studies was not possible.

Retrospective chart audits, pre and post comparisons, interviews, surveys and video analyses were used to explore the significance of meal support from a patient, clinician and parent / carer perspective. Whilst most studies were conducted in inpatient settings, meal support was also examined in a mental health community service and the home environment. This suggests that a meal support intervention can potentially be used across inpatient and outpatient settings as well as the home environment. Patients predominately had a diagnosis of anorexia nervosa, and were adolescents, however meal support was also utilised forbulimia nervosa and ARFID in children and adults. Meal support intervention is potentially suitable and beneficial for patients of all age groups and can be applied for a range of eating disorder diagnoses. Two studies that included patients who did not meet the diagnostic threshold for a diagnosis, found a beneficial impact of meal support on patient outcomes [35, 36], indicating the possible use of meal support as a preventive measure. Meal support was provided by clinicians from multi-professional backgrounds and in one study parents / carers were upskilled to deliver the intervention. Hence meal support could be conceptualised as a generic intervention, i.e., an intervention that could potentially be delivered by anyone, involved in the care of the patient, with adequate training.

In inpatient units, staffing levels available for the delivery of a meal support intervention varied significantly between studies. Practical and interpersonal aspects of the meal support intervention were explored and evaluated. Interpersonal aspects of meal support included: monitoring food intake, providing mealtime instruction, motivating and encouraging patients to complete the meal, expressing support and understanding, and providing psychoeducation [37].The included studies mainly focused on meal support within an inpatient setting. Supervised meals are assumed to be standard practice within specialised disorder inpatient units, and are considered best practice in facilitating refeeding in patients with eating disorders [42], however implementation across various settings has not been adequately researched. Our findings identified mixed results in terms of weight gain, length of stay, and future admissions, indicating that further research into this area is necessary. A complete lack of quantitative findings in adult groups means that findings of children and adolescents is potnentially being extrapolated to adult populations that may not be generalisable. Further studies into the benefits of meal support in adult populations is particularly warranted.

There was a lack of literature on provision of meal support in community settings or by parents and carers in the home environment; only three studies were conducted at home [34, 39, 41], with only two of these having meal support provided by the patients’ parents or carers [34, 41]. It has been identified as essential that meals should reflect ‘normal’ situations, to facilitate a positive transition back to regular eating habits [39, 40] Therefore, further research is needed to understand how meal support can be used in this way and how the intervention can be adapted and used in the transition from inpatient settings to both community and home environments. In the two studies where parents and carers provided meal support at home [34, 41], it was emphasised that provision of training and support resources were required for them to be able to adequately deliver meal support. Parents appreciated an intensive, tailored training approach that provided them with the skills needed to support their child [43]. Similarly, a meal support manual and DVD resource were rated favourably by parents, who reported they provide empowerment and the ability to implement empathic emotional and practical meal support at home [41]. However, further exploration of access to continued support in their use and implementation, as well as avenues for troubleshooting, would help provide a more robust framework to which the intervention is based and practiced. The review also revealed that each setting delivered meal support differently, however all studies reported that the intervention generally consisted of supervised eating followed by a rest period, with staff providing emotional and practical support throughout.

The evidence examined in this review shows that there is currently no agreed standardised, manualised, consistent approach to meal support available. This has been highlighted as problematic and being distressing for patients, carers and clinicians. Uncertainty and variation in the intervention provided across different environments has the potential to affect treatment outcomes and lead to inconsistencies in approach. It also increases missed opportunities to provide valuable interventions to people across various settings and in some ways could lead to negatives experiences and a change in the trajectory of the participants recovery journey. Developing a manualised meal support approach, co-designed with patients, caregivers, and healthcare clinicians is vital in integrating the experiences of those involved in the intervention.

Furthermore, significant variation in the outcomes measured in each of these studies was observed; including recovery outcomes (e.g., weight gain, length of stay, food consumption), experience outcomes, and satisfaction outcomes. In some studies, these outcomes were measured using non-validated, unstandardised measures. Inconsistency for key recovery outcomes were reported; for instance Kells et al. [35] reported a mean weight increase following meal support, whilst Couturier et al. [15] observed no change. Hence the clinical impact of meal support as an intervention is not yet clearly established.

### Limitations

This review synthesised the existing literature on meal support as an intervention for those with eating disorders and several limitations could be identified. The included studies were heterogenous in methodology and scope, which prevented the use of a meta-analysis to compare results across studies. Given that the literature on meal support is currently limited, none of the included studies were randomised controlled trials, which are the gold standard in assessing effectiveness of an intervention.

In a limited number of studies, the parent or carer of the patient, delivered the meal support intervention. Given that meal support is aligned with the person’s progress, and not the setting it is delivered in, further research involving caregivers would be beneficial in supporting recovery post-hospital discharge.

Although patients with a variety of eating disorder diagnoses were included in the studies, there is a need for further examination of how meal support intervention could be used and adapted for each diagnosis. In several of the studies, key demographic and clinical information, such as patients’ age and BMI was not reported. Reporting these variables is essential in understanding the patient population for which meal support might be suitable. However, these data were predominantly missing from papers focussed on describing the characteristics of the service, and not on patient outcomes. We therefore did not reach out to individual authors to request missing information. We acknowledge that failing to do so was another limitation of the study.

### Clinical implications

Due to the lack of evidence, meal support is rarely referenced in guidelines. More rigorously designed studies are required to ascertain its potential in the field eating disorder treatment. Meal support intervention as a standalone treatment is unique as it is based on a pragmatic, as opposed to a systemic, approach. It focuses solely on practical skills required during mealtime and can potentially be used across all ages. Delivery of the meal support intervention is anticipated to be fluid, and adapted in keeping with the patient’s recovery journey. For instance, the intervention might be directive in the initial phase of recovery, with the person providing meal support taking on a deliberate authoritative approach. However, as recovery progresses the style of support is anticipated to transition to a more collaborative approach in line with the patients increasing ability to make healthy choices over food intake. This is in keeping with existing models, in which advice on meal management is embedded in either systemic or cognitive behavioural concepts. In a family environment, the nature of meal time interactions are reported as imperative to recovery outcomes and family mealtime interactions commonly consist of both direct and indirect eating prompts and the provision of information, incentivising eating [44]. Training parents and carers in meal support can foster these interactions and provide the practical skills required to enable continued care at home outside of the inpatient setting.

Whilst this intervention might potentially be beneficial in treating eating disorders, it is essential to improve the understanding of individual differences, interpersonal components, environmental factors and how practical support is best delivered. Effectiveness may vary dependent upon where in the recovery process the patient is, as well as the level of family/caregiver support that the patient has, and social influences that are at play [41, 45]. Therefore, whilst the proposed manualised approach to meal support should outline a consistent assessment of needs and method for delivering the intervention, meal support approaches also need to be flexible and tailored to the needs of the patient and their available family and personal supports.

It is of note, that meal support is an important aspect of clinical care not only for patients with eating disorders. Meal support models outside the field of eating disorders might provide valuable insights on transferable skills, and concepts to meal support intervention. For instance, research into provision of meal support for patients with dementia identified core attitudes to its delivery that are potentially applicable and valuable for patients with eating disorders: i.e., the support person being able to establish a core connection, tailoring the intervention to the needs of the patient, whilst being receptive to the idea that needs may change [46] and recognising that good mealtime care helps patients to be empowered, and enables carers to respond in a way that encourages (but does not coerce) the patient to eat more [47]. However, due to the food-related distress observed in the disordered eating population, mealtime interventions are often seen as coercive, and it is uncertain how non-coercive, encouraging meal support could be implemented or whether it would be useful. This may be a differentiating factor between age cohorts that has not yet been investigated. Another example is the Altered Eating Framework, developed collaboratively with cancer survivors, to support disordered eating in cancer patients. It conceptualises seven core domains for assessment and meal support provision: physical anatomical, physical functional, sensory, behavioural, cognitive, cultural/social and emotional. This framework is an example for engaging in patient co-design to develop a comprehensive approach to meet clinical needs of a specific patient group while the outcome demonstrates potential for broader application. In addition, valuable insights for the development of a standalone meal support intervention could potentially be drawn from learnings and experiences gathered delivering the family meal as part of family therapy.

### Suggestions for future research

Most studies were conducted in AN, with only one study in ARFID that did find beneficial results. The differences between these diagnostic groups in terms of their benefit from meal support interventions should be further investigated. Cost effectiveness analyses are also missing from the literature, which may elucidate further evidence to support or rebuke the use of meal support strategies in inpatient settings. We also recommend the development and evaluation of a multidisciplinary and lived experience co-designed framework for a standardised, yet adaptable, manualised approach for meal support interventions.

## Conclusion

This systematic review has synthesised the current literature on meal support intervention for eating disorders. Studies have highlighted the benefits of meal support in facilitating recovery, however a number of gaps and opportunities for improvement are noted. The studies examined highlight the need for a framework and manualised approach to meal support intervention.

### Electronic supplementary material

Below is the link to the electronic supplementary material.

Supplementary Material 1

## Data Availability

Not applicable.

## Abbreviations

MBFT: Maudsley Family Based Therapy
FBT: Family Based Therapy
MFT: Multifamily Therapy
ARFID: Avoidant/restrictive food intake disorder
MMAT: Mixed-Methods Appraisal Tool
BMI: Body Mass Index
EDNOS: Eating Disorder Not Otherwise Specified
