# Supplementary material for: Meal support intervention for eating disorders: a mixed-methods systematic review
Source: J Eat Disord. 2024 Apr 22;12:47. doi: 10.1186/s40337-024-01002-2 (PMC11034063; doi:10.1186/s40337-024-01002-2)
Supplement: Supplementary file 1 — Supplementary Material 1 [file 40337_2024_1002_MOESM1_ESM.docx]

**Supplementary Table 1** McMaster Quantitative Quality Appraisal for Included Quantitative Studies

| Quantitative Studies | 1. Couturier and Mahmood (2009) | 2. Kells et al. (2013) | 3. Kells et al. (2017) | 4. Taylor et al. (2021) |
| --- | --- | --- | --- | --- |
| 1. Was the purpose stated clearly? | Yes | Yes | Yes | Yes |
| 2. Was relevant background literature reviewed? | Yes | Yes | Yes | Yes |
| 3. Design type | Cross sectional | Cohort study | Cohort study | Case control |
| 4. Was the sample described in detail? | Yes | Yes | Yes | Yes |
| 5. Was the sample size justified? | No | Yes | Yes | No |
| 5. Were the outcome measures reliable? | Yes | unclear | Yes | Yes |
| 6. Were the outcome measures valid? | Yes | unclear | unclear | Yes |
| 7. Intervention was described in detail? | Yes | No | No | No |
| 8. Contamination was avoided? | No | unclear | unclear | unclear |
| 9. Cointervention was avoided? | Yes | unclear | unclear | Yes |
| 10. Results were reported in terms of statistical significance? | Yes | Yes | Yes | Yes |
| 11. Were the analysis methods appropriate? | Yes | Yes | Yes | Yes |
| 12. Clinical importance was reported? | Yes | Yes | Yes | Yes |
| 13. Dropouts were reported? | unclear | No | No | Yes |
| 14. Conclusions were appropriate given study methods and results? | Yes | Yes | Yes | Yes |

**Supplementary Table 2** McMaster Qualitative Quality Appraisal for Included Qualitative Studies

| Qualitative Studies | 1. Beukers et al. (2015) | 2. Hage et al. (2015) | 3. Long et al. (2012a) | 4. Watt & Dickens (2018) |
| --- | --- | --- | --- | --- |
| 1. Was the purpose and/or research question stated clearly? | Yes | Yes | Yes | Yes |
| 2. Was the relevant background literature reviewed? | Yes | Yes | Yes | Yes |
| 3. What was the design? | Phenomenology | Phenomenology | Phenomenology | Phenomenology |
| 4. Was a theoretical perspective identified? | No | No | No | Yes |
| 5. Methods used? | Participant observation | Participant observation | Interviews | Interviews |
| 6. Was the process of purposeful selection described? | Yes | Yes | Yes | Yes |
| 7. Was sampling done until redundancy in data was reached? | Yes | unclear | unclear | unclear |
| 8. Was informed consent obtained? | Yes | Yes | Yes | unclear |
| 9. Clear and complete description of site? | Yes | Yes | Yes | Yes |
| 10. Clear and complete description of participants? | No | Yes | Yes | Yes |
| 11. Role of researcher and relationship with participants? | No | Yes | Yes | No |
| 12. Identification of assumptions and biases of the researcher | Yes | Yes | No | No |
| 13. Procedural rigor was used in data collection strategies? | Yes | Yes | Yes | Yes |
| 14. Data analyses were inductive? | Yes | Yes | Yes | Yes |
| 15. Findings were consistent with and reflective of data? | Yes | Yes | Yes | Yes |
| 16. Decision trail developed? | Yes | Yes | unclear | unclear |
| 17. Process of analysing the data was described adequately? | Yes | Yes | unclear | Yes |
| 18. Did a meaningful picture of the phenomenon under study emerge? | Yes | Yes | Yes | Yes |
| 19. Was there evidence of credibility? | Yes | Yes | Yes | Yes |
| 20. Was there evidence of transferability? | Yes | Yes | Yes | Yes |
| 21. Was there evidence of dependability? | Yes | Yes | Yes | Yes |
| 22. Was there evidence of confirmability? | Yes | Yes | Yes | Yes |
| 23. Conclusions were appropriate given the study findings? | Yes | Yes | Yes | Yes |
| 24. The findings contributed to theory development and future practice/research? | Yes | Yes | Yes | Yes |

**Supplementary Table 3** Mixed-Methods Quality Appraisal Tool for Mixed Methods Studies

| Mixed-Methods Studies | 1. Cairns et al. (2007) | 2. Long et al. (2012b) |
| --- | --- | --- |
| S1. Are there clear research questions? | Yes | Yes |
| S2. Do the collected data allow to address the research questions? | Yes | Yes |
| 5.1 Is there an adequate rationale for using a mixed methods design to address the research question? | Yes | Yes |
| 5.2 Are the different components of the study effectively integrated to answer the research question? | Yes | Yes |
| 5.3 Are the outputs of the integration of qualitative and quantitative components adequately interpreted? | unclear | Yes |
| 5.4 Are divergences and inconsistencies between quantitative and qualitative results adequately addressed? | No | Yes |
| 5.5 Do the different components of the study adhere to the quality criteria of each tradition of the methods involved? | Yes | Yes |
